# Thermoplastic Elastomeric Composites Filled with Lignocellulose Bioadditives. Part 1: Morphology, Processing, Thermal and Rheological Properties

**DOI:** 10.3390/ma13071598

**Published:** 2020-04-01

**Authors:** Justyna Miedzianowska, Marcin Masłowski, Krzysztof Strzelec

**Affiliations:** Institute of Polymer & Dye Technology, Lodz University of Technology, Stefanowskiego 12/16, 90-924 Lodz, Poland; marcin.maslowski@p.lodz.pl (M.M.); krzysztof.strzelec@p.lodz.pl (K.S.)

**Keywords:** thermoplastic elastomer blends, straw, waste management, processing, morphology

## Abstract

Thermoplastic elastomer blends based on natural rubber (NR) and ethylene-vinyl acetate copolymer (EVA) with different weight ratios (30, 40, 50, 60 and 70 parts per hundred rubber (phr) of NR) and 10, 20 and 30 phr of straw were prepared and characterized. Current environmental problems were the motivation to produce this type of system, namely: the need to replace plastics at least partly with natural materials; increasing the amount of renewable raw materials and managing excess straw production. When using this bioadditive in traditional materials, the high processing temperature can be problematic, leading to the degradation of straw fibers. The solution can be polymer mixtures that are prepared at significantly lower temperatures. Scanning electron microscope (SEM) imaging was used to investigate the particle size of fibers and phase morphology of composites. Moreover, determination of the thermal properties of the filler and composites showed that the processing temperature used in the production of NR/EVA blends reduces the risk of degradation of the natural filler. Differential scanning calorimetry (DSC) was used to determine the thermal behavior of the filled composites. Finally, rheological tests of materials allow the determination of optimal processing parameters and properties of materials in dynamic conditions. The proposed blends exhibit elastic properties, and due to the lack of chemical cross-linking they can be processed and recycled like thermoplastics. In addition, they offset the disadvantages and combine the advantages of natural rubber and ethylene-vinyl acetate copolymer in the form of thermoplastic elastomeric biocomposites.

## 1. Introduction

Thermoplastic elastomers (TPEs) are materials that are attracting growing interest from the scientific community. Within a certain temperature range, these materials combine the properties of crosslinked rubbers with the possibility of easy processing and recycling of thermoplastics [1]. They are characterized by phase micro-heterogeneity and domain morphology. The rigid part is formed by a phase with a higher glass transition/crystallization temperature (e.g., over 300 K), because below this temperature the segments of its macromolecules associate, and possibly crystallize. The domains of this phase act as thermo-reversible nodes of the network and particles of the reinforcing filler. Consequently, the material has a durable shape and is mechanically durable. The second phase has a lower glass transition temperature (usually less than 280 K), making it flexible and soft. This phase affects the material’s resistance to solvents, and determines chemical resistance and aging of the material [2]. The properties of all thermoplastic elastomers depend on the type, structure, dispersion and percentage content of both phases, the way they are connected, the nature and strength of interfacial interactions, as well as the presence of other substances that modify the properties of the phases or interactions between them [3]. 

A specialized group of materials formed by mixing natural rubber (NR) with thermoplastics in various proportions are the thermoplastic natural rubber blends (TPNRs). Thermoplastic polymers that could be used to prepare TPNRs include polystyrene (PS) [4], polypropylene (PP) [5], poly(methyl methacrylate) (PMMA) [6], and polyamide (PA) [7]. Another thermoplastic that can be blended with NR is ethylene-vinyl acetate copolymer (EVA), mainly because of its excellent properties (i.e., ozone, weather, solvent and flame resistance with improved mechanical properties). To date, several interesting papers have described mixing NR/EVA and testing the properties of the mixtures obtained. Koshy et al. studied blends of natural rubber and EVA to improve processing properties and resistance to thermal aging, γ radiation and ozone attack [8]. Sujith and Unnikrishnan conducted studies of the sorption of n-alkanes on crosslinked natural rubber/ethylene-vinyl acetate mixtures at various temperatures [9]. Zurina et al. presented the influence of radiation on tensile strength, dynamic mechanical, thermal and morphological properties of (epoxidized natural rubber) ENR/EVA mixtures [10].

One of the main directions of current research on TPE is the selection of a suitable filler that will provide the material with the desired properties. In the case of a combination of NR and EVA, it is also possible to try to use non-standard fillers to obtain blends with unique properties. An example of such a solution is the idea of adding biofillers in the form of lignocellulosic materials, one of which is cereal straw, to NR/EVA systems. This approach is relatively innovative, as according to the literature, there are few papers describing NR/EVA/bio-filler systems. The main problem raised in this type of research is the compatibility of the hydrophilic filler and the hydrophobic polymer matrix, but the very important aspect of flammability is usually overlooked. In addition, although only a small proportion of the available natural fibers have been tested, many of these, including straw, have application potential [11]. 

Straw consists of ripe or dried cereal, legumes, rapeseed and flax, which are characterized by a high dry matter content and high sorption capacity. Straw consists mainly of raw fiber and nitrogen-free compounds; it is made primarily of cellulose, hemicellulose and lignin [12]. Due to the huge amount of surplus straw produced at a global scale, it is necessary to manage this material properly. The basic method of managing straw is to use it as bedding material for animals. Straw is also used as a fertilizer because it is a source of organic matter and increases the amount of nutrients. Another way to utilize straw is to add it to feed [13,14]. Straw is a supplement to juicy fodder, gives a feeling of satiety and balances excess protein, but its nutritional value is low. It is inadvisable to burn straw after harvest, leading to environmental degradation [15]. This activity is used for weed control for soils, but results in almost complete biological deactivation [16]. Particular attention is paid to the use of straw for energy purposes, which is difficult to implement due to several significant technological and economic obstacles. An alternative use of straw is to apply it to products in the horticultural, industrial, construction and energy industries, and the key criterion determining the way it is used is usually the economic calculation [17]. All of these straw management methods have significant disadvantages, and the surpluses of straw produced are increasing. Therefore, intensive searching for alternative use of straw is underway. 

The authors’ earlier published works [18,19,20,21,22] show that use of such raw components as functional additives to typical elastomers is justified, but the idea of application to thermoplastic elastomers in the form of polymer blends may prove to be the most interesting proposal. In this article, the effect of lignocellulosic bioadditive content in the form of milled straw on morphology, processing, thermal and rheological properties of NR/EVA composites have been studied in detail. Composites with the addition of several biofiller amounts (10, 20 and 30 parts by weight) and different NR/EVA ratios (70/30, 60/40, 50/50, 40/60, 30/70) were prepared and their properties were examined with various techniques. Furthermore, the study of the obtained composites was preceded by characterization of cereal straw including its structural analysis and thermal stability.

## 2. Materials and Methods

### 2.1. Materials

- Polymer

Natural rubber (NR) RSS-I was provided by Torimex-Chemicals (Lodz, Poland). Natural, polymeric product was obtained from rubber milk secreted by *Hevea brasiliensis* trees, with a density of 0.930–0.988 g/cm^3^ at 20 °C and a decomposition temperature below 200 °C (data based on safety data sheet). 

Ethylene-vinyl acetate copolymer (EVA) known as EVA 1020 VN 5 with vinyl acetate content of 17.5%, a melt flow index of 2 g/10 min (190 °C, 2.160 kg) and density of 0.94 g/cm^3^ was supplied by Total Petrochemicals (Houston, TX, USA)

- Fillers

Cereal (wheat, oat, rye, barley and triticale) straw was collected from Polish farms. Straw was dried and next crushed using a Blixer 4 Robot Coupe (Vincennes, France)—grinding time of 20 min. Then sieve analysis was performed by using: a vibratory shaker with a set of sieves with 2.0, 1.0, 0.5, 0.25 mm nominal mesh size. In the following studies the fractions 0.5–0.25; >0.25 mm were ground using a Fritsch Pulverisette 5 Classic Line planetary ball mill (Idar-Oberstein, Germany) for 2 h, at a speed of 3000 rpm and a break of 30 min after 1 h. Detailed characteristics of straw as a filler were reported and studied in the authors’ previous works [19,23]. The compositions of NR/EVA and NR/EVA/straw blends are presented in Table 1. Each of the polymer mixtures was prepared in quantities such that NR and EVA constituted 100 parts per 100 parts rubber.

### 2.2. Methods

Elastomer mixtures, based on natural rubber/ethylene-vinyl acetate copolymer and straw (Table 1) were prepared using an internal mixer (Brabender, Duisburg, Germany) at 80 °C with 50 rpm. Process parameters, including temperature in the chamber and mixing energy, as well as the torque-time and temperature-time dependence, were recorded using the Brabender Mixer Software Program.

The polymer mixtures were formed using steel molds placed between the shelves of an electrically heated hydraulic press. The samples were pressed at 100 °C at 15 MPa pressure for 10 min.

The morphology of the filler and biocomposites was investigated by scanning electron microscopy (SEM). The SEM micrographs were obtained using a LEO 1530 Gemini scanning electron microscope (Zeiss, Oberkochen, Germany). The surface of reference blends was immersed in toluene for about 72 h to preferentially extract the NR phase. The samples were then dried in an air oven at 35 °C for 24 h. Prior to the measurement, composites were broken down using liquid nitrogen; their fractures were coated with carbon and examined.

The thermal stability (TG) of lignocellulose-based fillers and composites were studied using a thermogravimetric analyzer (Mettler Toledo, Greifensee, Switzerland). Samples of approximately 10 mg were placed in aluminum oxide crucible and heated from 25 °C to 600 °C in an argon atmosphere with a heating rate of 10 °C/min.

The thermal behavior of the composites was determined using a DSC analyzer (Mettler Toledo, Switzerland). Each sample (10 ± 0.2 mg) was first encapsulated in an aluminum closed pan. Next, the sample was cooled from 25 °C to −100 °C and then heated to 150 °C at a heating rate of 10 °C/min. All the experiments were carried out under a nitrogen atmosphere and the flow rate of gas was 80 mL/min. Prior to the measurements, the DSC analyzer was calibrated using indium and n-octane as standards. Liquid nitrogen was applied as a cooling agent.

Dynamic mechanical analysis (DMA) was performed based on changes in the dynamic moduli as a function of the oscillation strain using the same rotational rheometer with a plate-plate system (Ares G2 Rheometer, New Castle, NY, USA). The test parameters: strain: from 0.1% to 150%; sample deformation rate: 10 rad/s; test force: 5 N; temperature: 25 °C. The Payne effect (ΔG’) values of the composites were calculated based on Equation (1).

ΔG’ = G’min (lim10^−1^) − G’max (∞) (1)

where, G’min (lim 10^−1^) is a composite storage modulus determined under the deformation of 10^−1^%; G’max (∞) is a composite storage modulus determined under the maximum deformation.

Rheological properties were also tested using a rotational rheometer with a plate-plate system. The test parameters: strain: 1%; sample deformation rate: 628–0 rad/s; test force: 5 N; temperature: 80 °C. Based on the results obtained from the strain and time sweep experiments, the rheological behavior of the materials were studied in their linear viscoelastic regions.

## 3. Results and Discussion

### 3.1. Processing (Torque Development)

**Figure 1:**
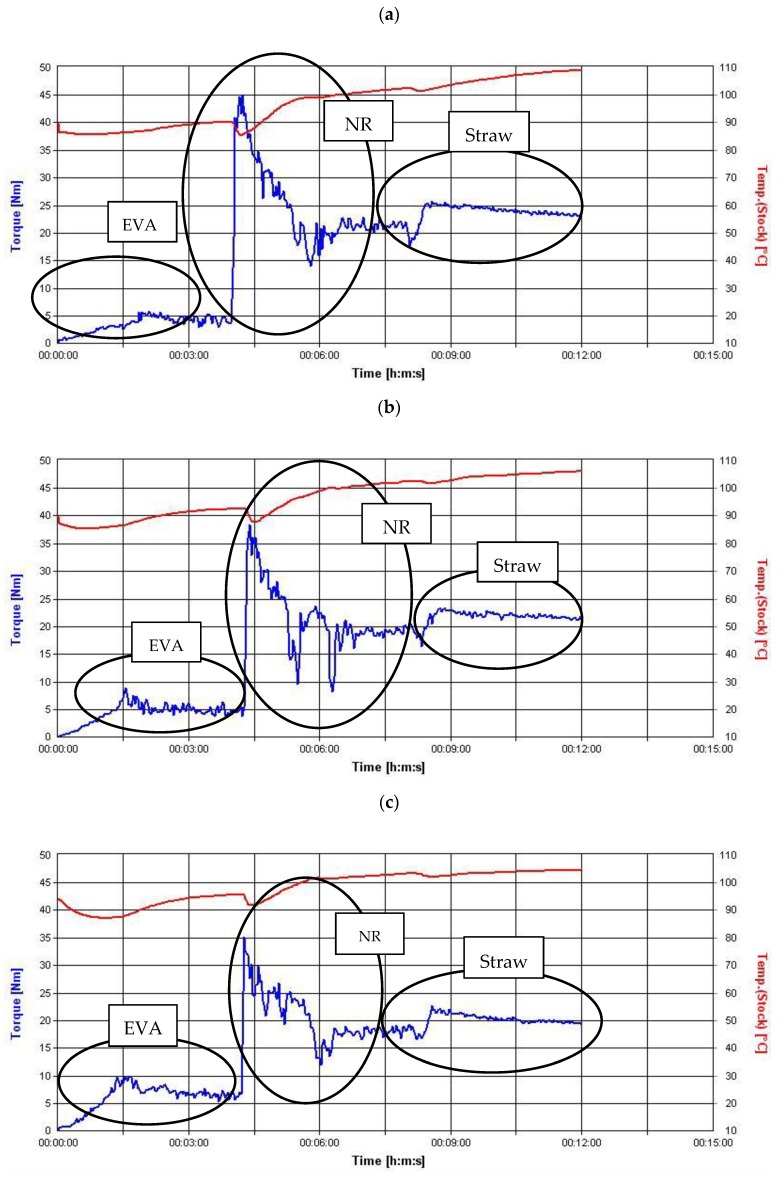

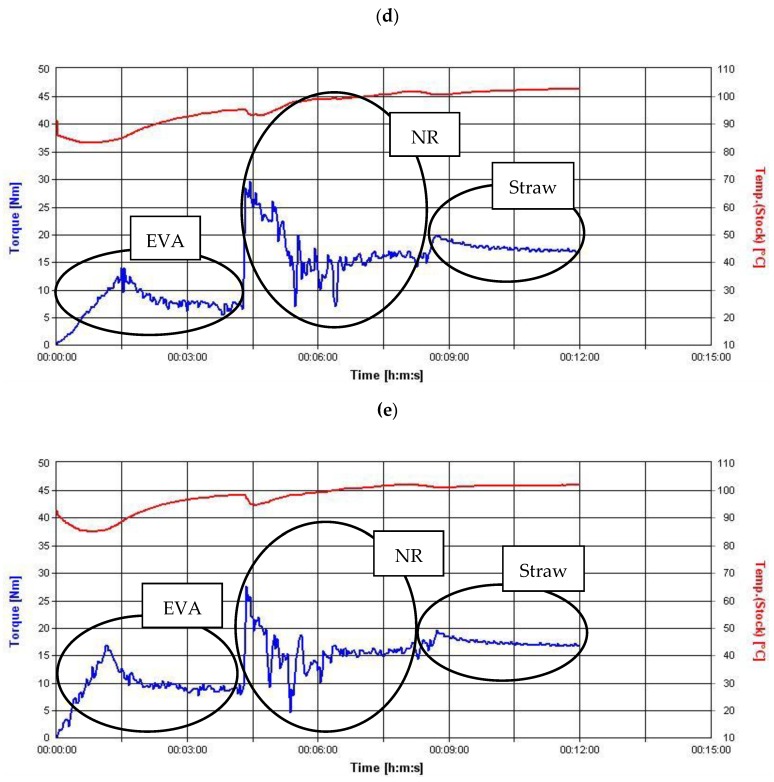
The torque-time and temperature-time curves of blends filled with 10 parts per hundred rubber (phr) straw: (**a**) 70/30/10 natural rubber (NR)/ethylene-vinyl acetate copolymer (EVA)/straw; (**b**) 60/40/10 NR/EVA/straw; (**c**) 50/50/10 NR/EVA/straw; (**d**) 40/60/10 NR/EVA/straw; (**e**) 30/70/10 NR/EVA/straw.

The torque-time curves were used to present the processability of the blends. The torque-time curves of NR/EVA blends, with lignocellulose filler (10 phr) at different blend ratios are shown in Figure 1. In general, all curves had three regions representing the loading of EVA, NR and straw, respectively.

The EVA copolymer was the first component introduced into the mixer and was plasticized for four minutes. The increase in torque resulting from its presence increased with the EVA volume share in the mixer chamber. After four minutes, natural rubber was added, causing the next increase in torque observed in the charts. As with the first component, the torque value depended on the amount of rubber added. After another three minutes, the rotor torque began to stabilize, and the natural rubber also became plasticized. In the eighth minute of mixing, straw was added in portions to the mixtures, which resulted in an another increase in the torque value. The moment recorded at the end of the mixing process (after 12 min) was greater the higher the rubber content of the blend. It probably resulted from the higher viscosity of the system and thus the work performed by the device rotors needed to mix the components.

**Figure 2:**
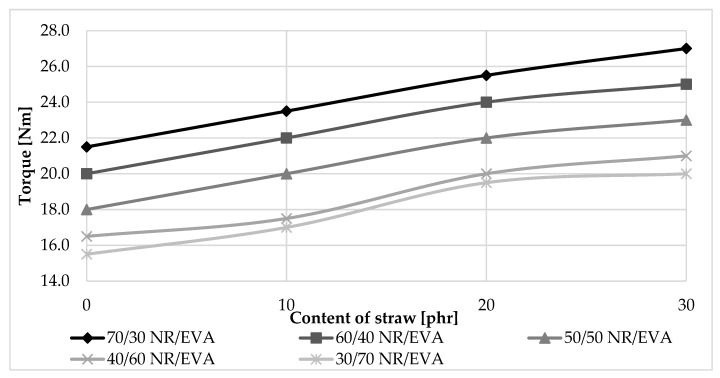
The impact of straw content on the torque value during blend preparation.

Straw increased the viscosity of the material. The amount of filler used also had an effect on the value of the generated torque (Figure 2). By comparison with the corresponding binary mixtures (NR/EVA), the torque values for the tricomponent blends shown in Figure 2 increased by about 2 Nm, 4 Nm and 5 Nm when straw content added was 10 phr, 20 phr and 30 phr, respectively. The advantage of composites containing less straw and more EVA is the smaller moment needed to plasticize the material, thus requiring lower machine exploitation and energy consumption, resulting in the potential for substantial savings to be obtained during high-volume production. In addition, as a result of reduced friction, the temperature of the entire system increased less rapidly and to a lower value, so that it was easier to control the process parameters. Focusing on processing properties, it should also be emphasized that the composites produced were prepared without the use of crosslinking agents. Such a solution, in contrast to vulcanization, i.e., the standard technology used in elastomeric materials and their blends [24,25], has many benefits. The advantage of all prepared compositions over vulcanizates is a huge saving of time, energy, equipment and human resources. This eliminates the need for quality control of composites before and after vulcanization. The process temperature is up to 100 °C lower than the vulcanization temperature, which is, again, a source of great energy savings, and facilitates process control. It is also worth noting that the presented composites can be recycled and used in material recycling processes, which is particularly valuable from the point of view of sustainable development principles.

### 3.2. Morphology

**Figure 3:**
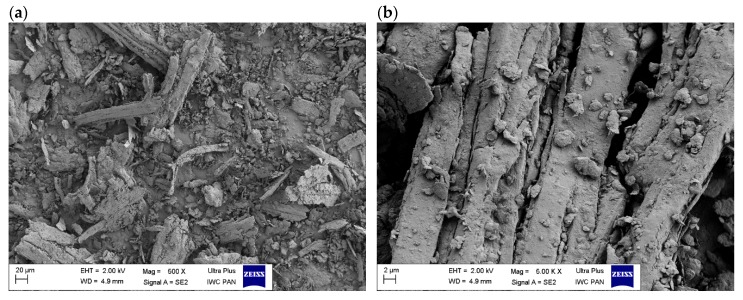
SEM images of ground straw under two magnifications: (**a**) 500× (**b**) 5000×.

SEM images (Figure 3) show the structure of straw crushed to micrometric dimensions in a ball mill. The purpose of grinding was not only to reduce the size of the straw particles, but also to break down the cell walls and release some individual components. In addition, the fibers have been shortened by milling process. The image at 500× magnification shows that the straw was fibrous, most of the elements are elongated and are in parallel bands of varying length and width. Nevertheless, the material obtained revealed a large nonhomogeneity concerning both size and shape of the structural particles. For example, in the 5000× image, numerous fine straw particles of irregular shape could be observed on the fiber surface.

**Figure 4:**
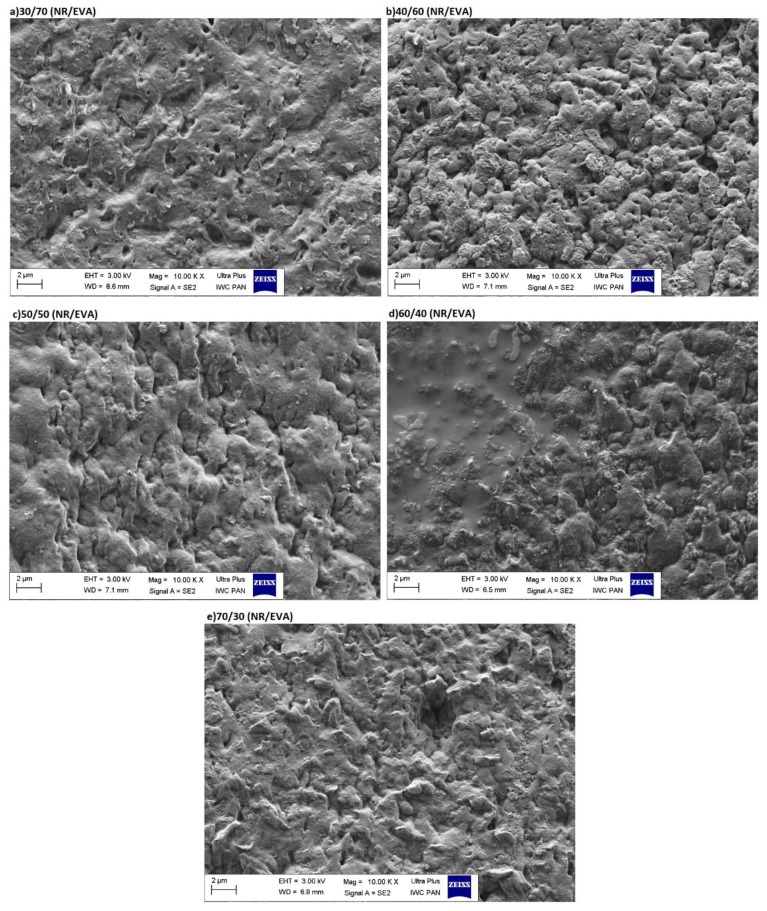
Morphology of NR/EVA reference blends (previously soaked in toluene).

SEM images in Figure 4a–e show the structure of reference, filler-free, composites of NR/EVA after toluene treatment. Based on these SEM images, it can be seen that a continuous structure was achieved in all NR/EVA ratios in this case. The lack of visible delamination and the interpenetrating but not mixing phases of the composites lead to the conclusion that both elastic and thermoplastic properties could be expected. Appropriate morphology is a precondition for obtaining a material showing both elastic and thermoplastic properties. A dispersed rubber system in a rigid plastomer would have increased flexibility and impact strength. On the other hand, if the material had the opposite structure, i.e., a plastomer dispersed in the rubber matrix, the desired properties would not be obtained either. The material would then be soft, but would deform irreversibly under load and would have low strength. However, the simultaneous combination of elastic and thermoplastic properties is only possible for physically micro-homogeneous mixtures of rubber with a plastomer with co-continuous two-phase morphology, i.e., interpenetrating phases [26]. A mixture with co-continuous morphology is possible if the viscosity of the components is relatively low and close to each other, and the individual phases are mixed in an appropriate volume.

**Figure 5:**
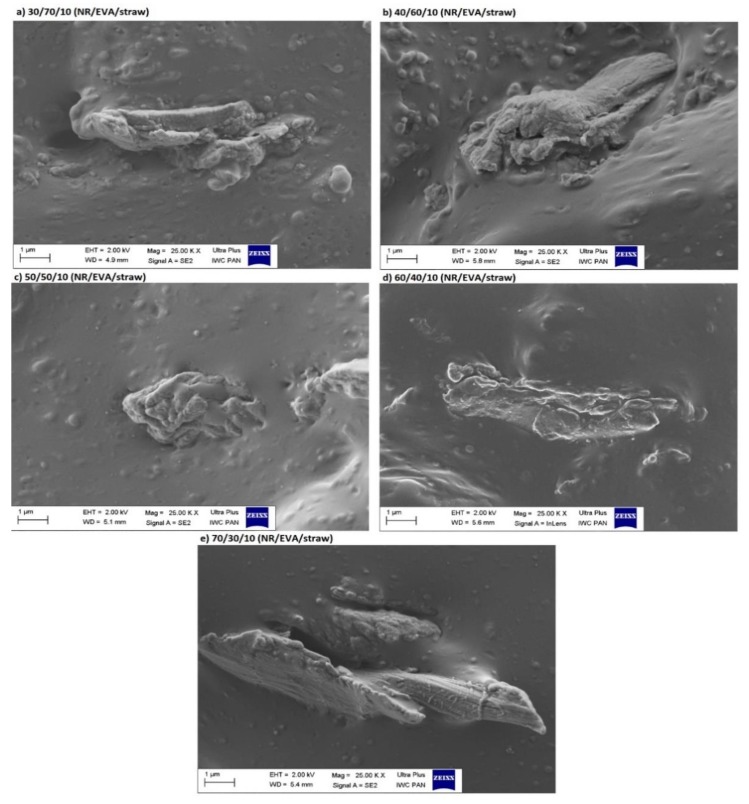
SEM micrographs of NR/EVA/straw composites filled with 10 phr of straw.

Figure 5 presents SEM photos of composites filled with 10 phr straw. Based on their analysis, individual straw fibers were observed, with no straw particles aggregated into larger agglomerates. The degree of fiber dispersion was high, resulting in a homogeneous structure. Fibers were dispersed to a large extent, which allows a uniform structure to be obtained. As the content of the natural rubber blend increased, better coverage of the straw fiber by the polymer matrix probably occurred (Figure 5e), which may indicate a greater interaction of straw with natural rubber.

### 3.3. Differential Scanning Calorimetry (DSC) Analysis

**Figure 6:**
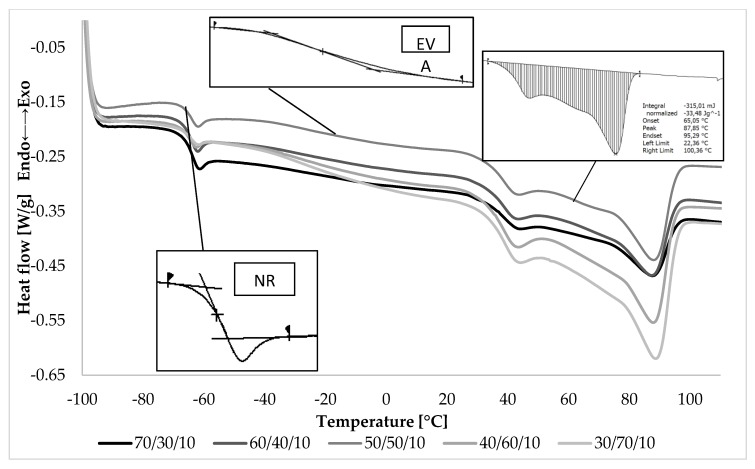
Differential scanning calorimetry (DSC) graphs of NR/EVA/straw composites filled with 10 phr of straw.

Based on the DSC thermograms taken of blends containing 10 phr staw (Figure 6), the following parameters were determined: glass transition temperatures (T_g_), melting peak temperatures (T_mp_) and the enthalpy of fusion (ΔH_f_) of materials. In the low-temperature region of the DSC curves, there were two inflection points, so the samples were characterized by two glass transition temperatures (Table 2). The first inflection point (about −66 °C) corresponds to the glass transition temperature of the NR phase, and the second (about −36 °C) to the glass transition temperature of the EVA phase. Obtaining of two glass transition temperatures indicated that in the case of NR/EVA blends containing straw, one of the criteria of polymer miscibility was not achieved. However, the lack of miscibility in the thermodynamic sense did not yet indicate a lack of compatibility. Compatibility is the ability of two different polymer phases to coexist in a mixture in separate, dispersed phases, which may lead to the combined properties of each of them in the material as a whole. Often, such a system is called partially miscible. In all the systems studied, every single DSC thermogram shows two glass transition temperatures corresponding to the two polymer phases—NR and EVA copolymer [1]. SEM images showing the morphology of co-continuous interpenetrating phases testify to the intermediate compatibility of the components of the tested blends.

In the area of high-temperature thermograms, the endothermic effect was visible, resulting from melting of the crystalline phase. The melting process of the crystalline phase in the blends began at a temperature of about 30 °C and ended at 100 °C. It can be considered as a fusion process involving the melting of the crystalline phase contained in natural rubber and EVA copolymer. Within these temperatures, there could also be a thermal effect associated with the evaporation of moisture contained in the lignocellulosic material (at about 90–100 °C). Nevertheless, the melting of the crystalline phase of the EVA copolymer had the greatest impact on this phenomenon.

Maximum melting peak temperature (T_mp_) shifted towards higher temperatures as the weight fraction of crystallizing EVA copolymer increased. Increasing the EVA copolymer content in blends caused an increase in the content of the crystalline phase, and thus an increase in melting heat that was confirmed by higher values of the enthalpy of fusion (ΔH_f_) (Table 2).

### 3.4. Thermal Stability

**Figure 7:**
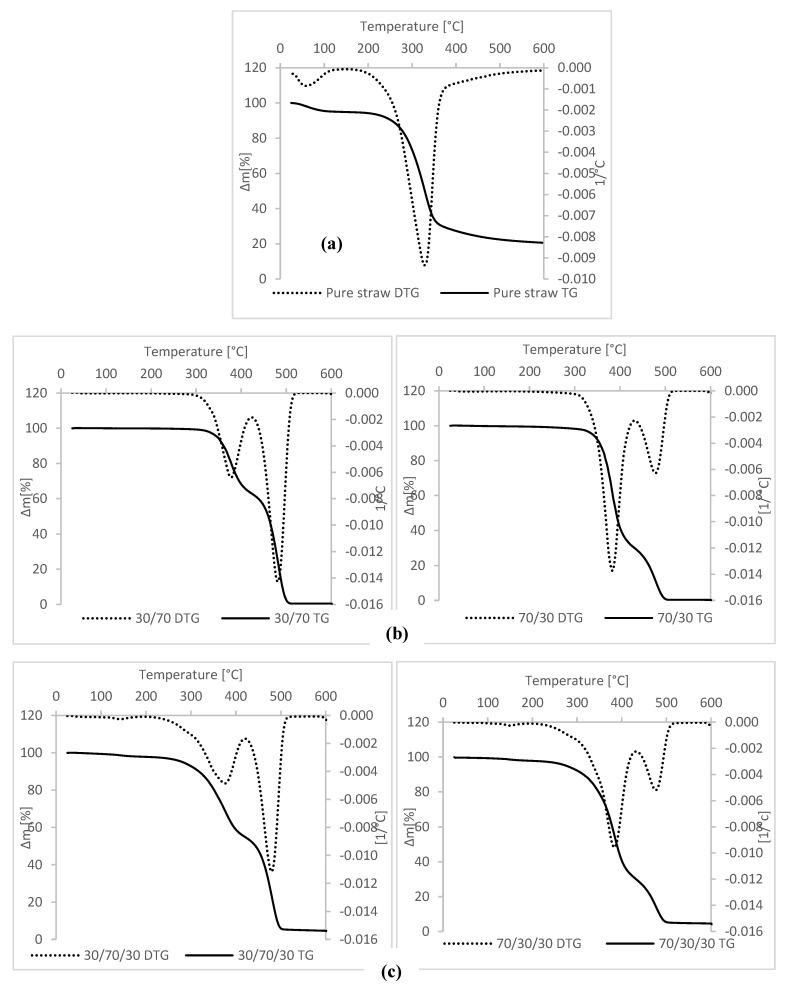
The derivative thermogravimetry (DTG) curves of: (**a**) pure straw (**b**) 30/70 and 70/30 blends of NR/EVA and (**c**) 30/70/10 and 70/30/30 blends of NR/EVA/straw.

The thermal stability of the fillers used in polymer composites plays a key role in their processing capabilities. When using plant fibers with generally low thermal resistance, their decomposition temperature usually determines the processing temperature of composites filled with lignocellulosic additive. Thermogravimetric analysis of lignocellulosic material derived from straw is presented in Figure 7a. The derivative thermogravimetry (DTG) curve was characterized by two peaks. The first stage of degradation of the filler occurred in the temperature range of 28–100 °C and was associated with the removal of moisture contained in the sample. Straw as a hygroscopic material has a high ability to absorb moisture from the air, although the material was dried and conditioned before measurement. This phenomenon was unavoidable due to non-removable bound water retained into the straw matrix even after its drying [27]. The weight loss associated with this process was about 5%. A similar effect was reported by Hornsby et. al. [28]. The onset of the main peak on the DTG pure straw curve is located at 172 °C and the peak maximum at 327 °C. Intensive decomposition of lignocellulosic material was observed up to 380 °C. At this stage, the amount of released volatile was mainly due to the thermal decomposition of hemicellulose and cellulose, and partly of lignin. At 380 °C, the weight loss was 72%. However, the permanent residue after the pyrolysis process was 20%, and it consisted mainly of mineral substances and ash [29].

The low thermal stability of pure straw (Figure 7a) may limit its use in plastic-based composites where high processing temperatures are required. Prolonged exposure of lignocellulosic material to elevated temperatures causes its degradation and contributes to the loss of material properties. However, the thermoplastic elastomer in the form of NR/EVA blends has a low processing temperature. At 80 °C the system has already become plasticized and the forming process is possible. This processing temperature range ensures that straw can be used as a filler of blend without exposing it to thermal degradation.

Using thermogravimetric analysis, the thermal stability of the obtained systems, both unfilled NR/EVA blends and straw-containing composites, was also examined. The test results for 30/70 and 70/30 NR/EVA samples are presented in Figure 7b, and thermograms for composites filled with 30 phr straw are included in Figure 7c. For reference samples without filler, two peaks associated with blended components were observed on the DTG curves. The first of these was ascribed to degradation of natural rubber and the vinyl portion of EVA copolymer. Natural rubber decomposes into a single degradation step at an onset temperature of approximately 320 °C [30]. Pure EVA shows double degradation stages with an onset temperature at 335 °C in the first stage, which corresponds to the degradation of vinyl acetate moieties with the release of acetic acid. The second degradation step was related to the polyethylene chain degradation with an onset degradation temperature of 435 °C [24]. Accordingly, the second stage at higher temperature observed on the DTG curve (Figure 7b) was assigned to the decomposition of hydrocarbon chains. The addition of straw to blends caused a decrease in the thermal stability of the composites. There are three peaks on the DTG curves of blends with filler (filled blends). The first one appears at a temperature of about 100 °C and corresponds to the evaporation of moisture. This phenomenon occurs much later than in the case of the results obtained during the characteristics of the filler itself—pure straw. This is the result of limited diffusion of water vapor through the sample, which causes this process to shift. The next stage of biocomposite decomposition begins at 210 °C, and, in the case of filled composites, it consists of the decomposition of lignocellulosic material, natural rubber and degradation of poly(vinyl acetate) segments in the EVA. The third intense peak on the DTG curve with a peak temperature of ca. 480 °C corresponds to the degradation of the polyethylene segments in the EVA. The process of pyrolysis of unfilled blends was almost complete, the solid residue after analysis being about 0.5%. In the case of composites containing straw, the quantity of solid residue was about 5% after filler degradation was noted.

### 3.5. Dynamic Mechanical Properties as a Function of Deformation Amplitude

**Figure 8:**
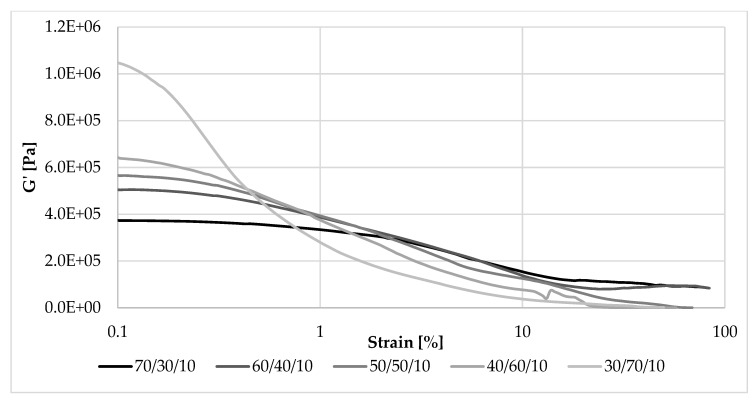
Dependence of the storage modulus on the strain amplitude and the NR/EVA ratio at 10 phr straw filling.

**Figure 9:**
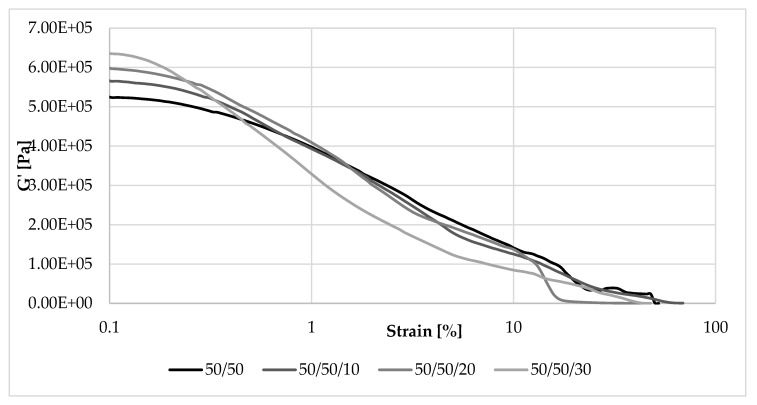
Dependence of the storage modulus on the deformation amplitude and straw content for composites with the ratio NR/EVA 50/50.

**Figure 10:**
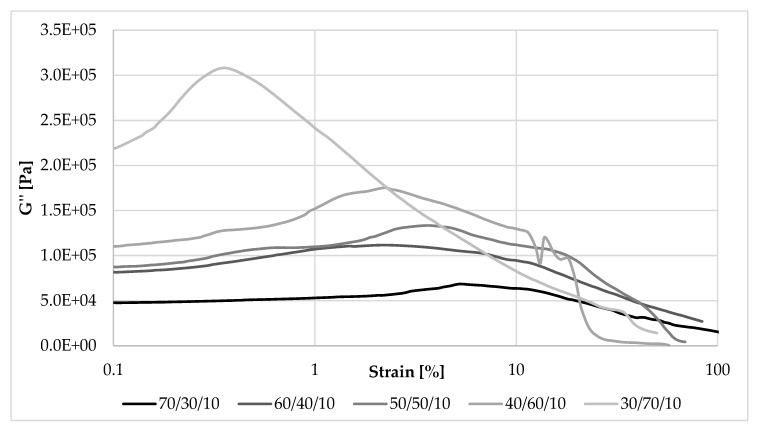
Dependence of the loss modulus on the deformation amplitude and the NR/EVA ratio at 10 phr straw filling.

**Figure 11:**
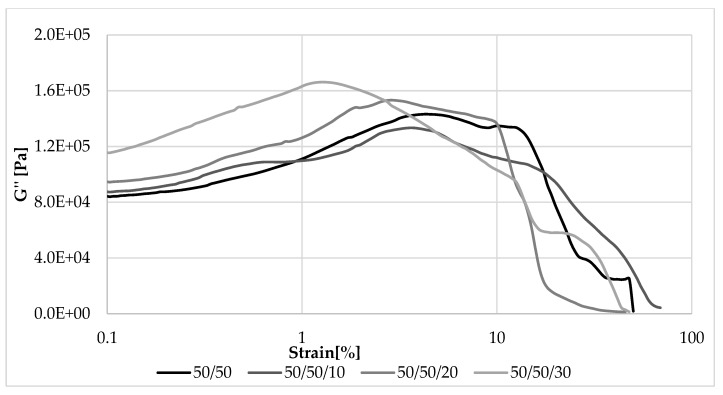
Dependence of the loss modulus on the amplitude of deformation and straw content for composites with a NR/EVA ratio of 50/50.

Figure 8, Figure 9, Figure 10 and Figure 11 show changes in storage and loss modulus as a function of deformation at a constant sample deformation rate of 10 rad/s.

#### 3.5.1. The Storage Modulus

As can be seen in Figure 8, among the composites containing 10 phr of straw, at the smallest oscillatory amplitudes of strain exerted on them, the largest storage (elastic) modulus was demonstrated by a sample with the NR/EVA ratio of 30/70 (G’max = 1.1 MPa). The sample containing the same amount of straw, but NR and EVA in the opposite proportion exhibited the smallest storage modulus: G’max of 0.37 MPa. This relationship reversed very quickly, with a deformation amplitude of less than 2%. During the deformation of the sample, the secondary structure of the filler (filler-filler and filler-polymer interactions) was destroyed and the storage modulus was rapidly reduced—the decrease was the greater, the greater its initial value. As a result of this phenomenon, in almost the entire range of applied strain amplitudes, a greater content of NR led to a higher storage modulus, and thus the ability to accumulate energy during deformation flexibility.

As shown in Figure 9, samples with the same NR/EVA ratio but containing straw exhibited the highest storage modulus at low strain. Initially the G’max vaule of the unfilled composite was 0.53 MPa and filled with 30 parts by weight, already 0.64 MPa. Thus, the more straw fibers in the composite, the stronger the filler-filler interaction, and the more extensive the “secondary structure”. As a result of increasing the amplitude of deformations, the “secondary structure” of the filler was destroyed, whereby the modulus decreased and eventually was larger for samples with a lower filler content.

#### 3.5.2. The Loss Modulus

Loss modulus is another parameter that was analyzed during measurements in dynamic conditions. Changes in its value as a function of deformation amplitude allowed characterization of the sticky properties of composites. The higher the content of EVA and straw, the higher the G” at strain amplitude up to about 10% (Figure 10 and Figure 11). In these cases, a larger amount of deformation work is dissipated as heat more easily than in samples containing more rubber. For this reason, less energy is stored in the material structure, the elasticity of the material is reduced, and sample recovery to its original shape is more difficult. The loss modulus is also dependent on the rate of destruction and reformation of the structure, conditioned by internal friction. At higher strain amplitudes, the dependence of G’ on the content of EVA and straw was reversed, similar to that of the storage modulus.

#### 3.5.3. Payne Effect

Determining the maximum and minimum values of the storage modulus (G’min, G’max) allowed calculation of the Payne effect (ΔG’)—the decreasing of the G’ modulus with increasing shear deformation amplitude (Table 3). The Payne effect occurs for active fillers and depends on a filler-filler interaction. The greater the strength of these interactions, the stronger the “secondary structure’’ and the greater the potential for strengthening the composite. This effect is not dependent on the nature of the medium, but if the filler-polymer interactions change, the straw fibers will form a different secondary structure. The study clearly showed that the more ethylene-vinyl acetate copolymer is present in the blend, the higher the Payne effect value. The filler-polymer interactions could be smaller than in the matrix with the higher content of NR.

The Payne effect is also dependent on the degree of dispersion of the filler particles. Its value will be high if the filler has clumped distributions of aggregates and agglomerates and low if they are well dispersed in the polymer matrix. The filler-filler interaction is dependent on chemical and physical interactions between particle surfaces, network morphology and filler volume. The higher Payne effect for samples containing more EVA testified to the greater straw aggregation and agglomeration in this matrix. Perhaps the filler-filler interactions prevailed over the filler-polymer interactions, and straw tended to combine their fibers into clusters. An appropriate balance of filler-filler and filler-polymer in the matrix with the advantage of NR allowed for better dispersion of the filler.

The strengthening effect of fillers is strictly dependent on the processes of energy dissipation during deformation. This, in turn, has an impact on tensile strength, abrasion and tearing, ability to damp vibrations, and fatigue strength of samples. Considering specific applications, energy dissipation is crucial for structural elements subjected to dynamic deformations, such as anti-vibration pads, springs and, above all, tires.

### 3.6. Dynamic Mechanical Properties as a Function of Deformation Frequency

**Figure 12:**
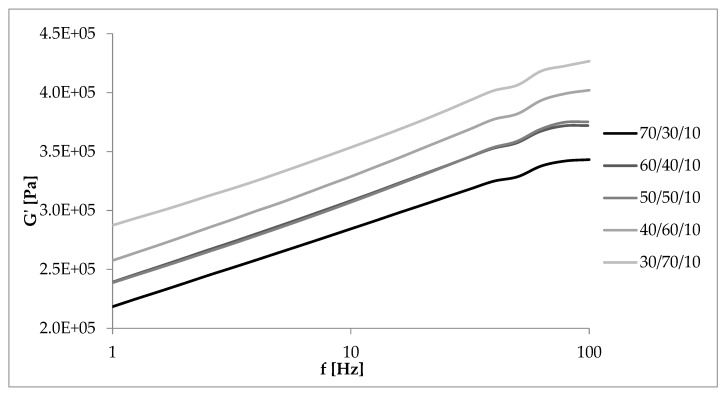
Dependence of the storage modulus on the frequency of deformations and the NR/EVA ratio at 10 phr straw filling.

**Figure 13:**
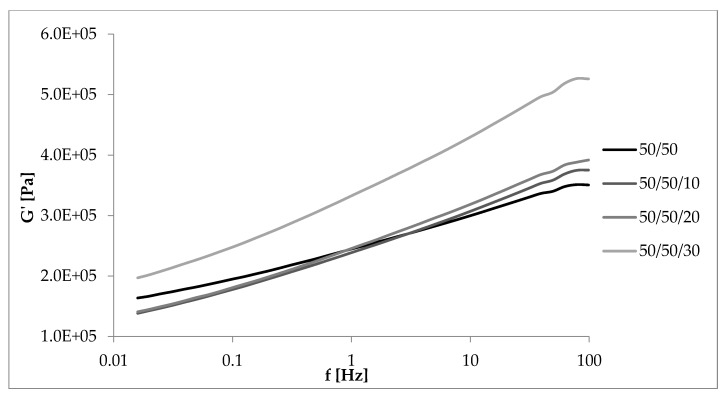
Dependence of the storage modulus on the frequency of deformations and straw content for composites with a NR/EVA ratio of 50/50.

**Figure 14:**
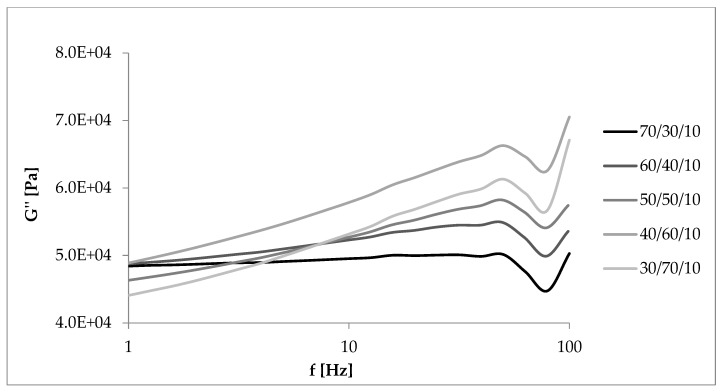
Dependence of the loss module on the deformation frequency and the NR/EVA ratio at 10 phr straw filling.

**Figure 15:**
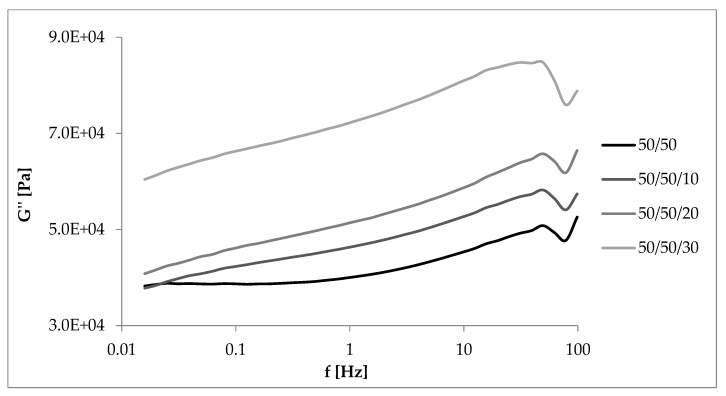
Dependence of the loss module on the frequency of deformations and straw content for composites with a NR/EVA ratio of 50/50.

Figure 12, Figure 13, Figure 14 and Figure 15 showed the dynamic rheological properties of composites with different NR/EVA ratios and different straw contents. Both the storage and loss moduli increased with increasing frequency as a result of the relaxation of polymer chains. The G’ values were greater than G” ones over the entire frequency range with no crossover points. It follows that the applied force was less than the interaction between the components and intramolecular forces. Composites displayed the ability to accumulate energy that could be used to return macromolecules to their original configuration [31,32]. Figure 13, Figure 14 and Figure 15 showed that the storage and loss modulus were higher for straw-filled composites compared to the no-filler blends. The larger the content of filler, the higher the moduli values (both G’ and G”), depending on the frequency of deformations. A particularly large difference is visible for samples with a NR/EVA/straw ratio of 50/50/30. G’max (with dependence of the storage modulus on the frequency) of a NR/EVA/straw ratio of 50/50/20 was about 0.39 MPa, and with the addition of another 10 parts by weight of straw increased to 0.53 MPa (Figure 13). For this reason, composites with straw were characterized by a greater ability to accumulate and dissipate energy under the influence of dynamic loads. The addition of fibers to the rubber matrix limited the mobility of polymer chains in the molten state by changing the response of the conservative module, leading to a different loss modulus than those of unfilled systems.

In summary, the conducted studies showed that it is possible to obtain highly flexible biocomposites from a combination of thermoplastic and elastomer materials filled with cereal straw. The advantage of such materials is the lack of the need for chemical crosslinking, which results in lowering the processing temperature, resulting in energy and economic savings. It also allows the reprocessing and recycling of such materials at the end of the product life cycle. In addition, such composites offset the disadvantages and combine the advantages of natural rubber and ethylene-vinyl acetate copolymer in the form of thermoplastic elastomeric biocomposites.

## 4. Conclusions

A promising compromise between environmental friendliness and performance are bio-blends, obtained by replacing part of petroleum-based polymers with natural additives. As a result of this research, new biocomposites were obtained which, according to the literature, have not yet been known, characterized and described. The polymer matrix was a thermoplastic elastomer in the form of a blend of natural rubber and ethylene-vinyl acetate copolymer, while the filler was ground cereal straw.

The processing and morphology properties, as well as thermal and rheological behaviors of the NR/EVA blend with different straw contents, were compared with their unfilled counterparts. The most important results are listed below:

(1) The torque recorded at the final stage of mixing increased with increasing rubber content in the blend, which is due to its higher viscosity and the need to overcome the greater resistance by the rotors in the device. The addition of straw increased the viscosity of the material and also had an impact on the value of the generated torque.
(2) Analysis of SEM images showed that the fragmented straw has a fibrous form, most of the elements are elongated and parallel bands of different length and width. Its fibers dispersed to a high degree in the polymer matrix.
(3) There are two inflection points in the low-temperature region of DSC curves, so the samples have two glass transition temperatures. Increasing the EVA content in blends resulted in an increase in the content of the crystalline phase, and thus an increase in melting heat.
(4) Thermogravimetric analysis of both the filler and composites showed that the processing temperature used in the production of NR/EVA blends reduces the risk of straw degradation.
(5) Storage and loss modules were higher for samples with higher EVA content and straw only for small deformation amplitudes. At the initial testing stage, higher modulus values for samples with a higher EVA content were probably due to the poorer straw dispersion in the copolymer than in the rubber
(6) While subjecting the samples to deformations of increasing frequency, the value of both storage and loss modules increased, which was the result of the relaxation of polymer chains. In the whole range tested, the G’ value was greater than G” and the module charts did not intersect. This leads to the conclusion that the force applied during the tests was less than the intermolecular forces and forces between fibers, and the composites had the ability to accumulate energy.
(7) The loss angle tangent showed the ratio of energy lost to the energy retained during cyclic deformation. In the low-frequency range, samples with a larger proportion of NR had higher tanδ, but at higher frequencies, the relationship reversed and the effect of elastic properties increased.

## Figures and Tables

**Table 1:** The composition of polymer mixtures.

Sample Name	Natural Rubber	Ethylene-(Vinyl Acetate)	Straw
	(phr *)		
**70/30**	70	30	0
**70/30/10**			10
**70/30/20**			20
**70/30/30**			30
**60/40**	60	40	0
**60/40/10**			10
**60/40/20**			20
**60/40/30**			30
**50/50**	50	50	0
**50/50/10**			10
**50/50/20**			20
**50/50/30**			30
**40/60**	40	60	0
**40/60/10**			10
**40/60/20**			20
**40/60/30**			30
**30/70**	30	70	0
**30/70/10**			10
**30/70/20**			20
**30/70/30**			30

* phr, parts per hundred rubber.

**Table 2:** Glass transition temperatures (T_g_), melting peak temperatures (T_mp_) and the enthalpy of fusion (ΔH_f_) of NR/EVA/straw blends.

Sample	T_mp_ (°C)	ΔH_f_ (J/g)	T_g_ (°C)	
**70/30/10**	87.05	21.35	−66.71	−31.38
**60/40/10**	87.30	28.51	−67.03	−40.53
**50/50/10**	87.85	33.48	−66.65	−35.52
**40/60/10**	87.89	42.28	−67.77	−36.83
**30/70/10**	88.47	46.15	−67.17	−38.00

**Table 3:** Storage and loss modules and the Payne effect of composites.

Sample Name	Content of Straw	G’min	G’max	G”max	ΔG’
[phr]	[phr]	[MPa]			
**70/30**	0	0.076	0.260	0.038	0.19
	10	0.073	0.370	0.068	0.30
	20	0.069	0.370	0.082	0.31
	30	0.002	0.460	0.090	0.46
**60/40**	0	0.092	0.260	0.044	0.17
	10	0.080	0.510	0.110	0.43
	20	0.003	0.710	0.160	0.71
	30	0.002	0.430	0.110	0.43
**50/50**	0	0.010	0.530	0.140	0.52
	10	0.010	0.570	0.130	0.56
	20	0.001	0.600	0.150	0.60
	30	0.001	0.640	0.170	0.64
**40/60**	0	0.020	0.660	0.170	0.64
	10	0.001	0.650	0.180	0.65
	20	0.016	0.780	0.250	0.77
	30	0.010	0.560	0.150	0.55
**30/70**	0	0.010	0.860	0.220	0.85
	10	0.018	1.100	0.310	1.08
	20	0.025	0.950	0.260	0.93
	30	0.010	1.100	0.340	1.09
